# Supplementary material for: Dynamical stability and mechanical impedance are optimized when manipulating uncertain dynamically complex objects
Source: PLoS Comput Biol. 2025 Dec 9;21(12):e1013797. doi: 10.1371/journal.pcbi.1013797 (PMC12704904; doi:10.1371/journal.pcbi.1013797)
Supplement: S1 Text — This first section in the supplementary information document reports forward simulation results like Fig 6D for all the pendulum conditions (Short, Medium and Long) and variables (Absolute Force, Force Smoothness, and Risk of Ball Escape) in Fig AA. The corresponding KL-Divergence results are also summarized in Fig AB. The next section reports in detail the cost and state weighting matrices that were used in the Stochastic Open-loop Optimal Control simulations. (PDF) [file pcbi.1013797.s001.pdf]

Forward Simulation and KL-Divergence Results for All Conditions

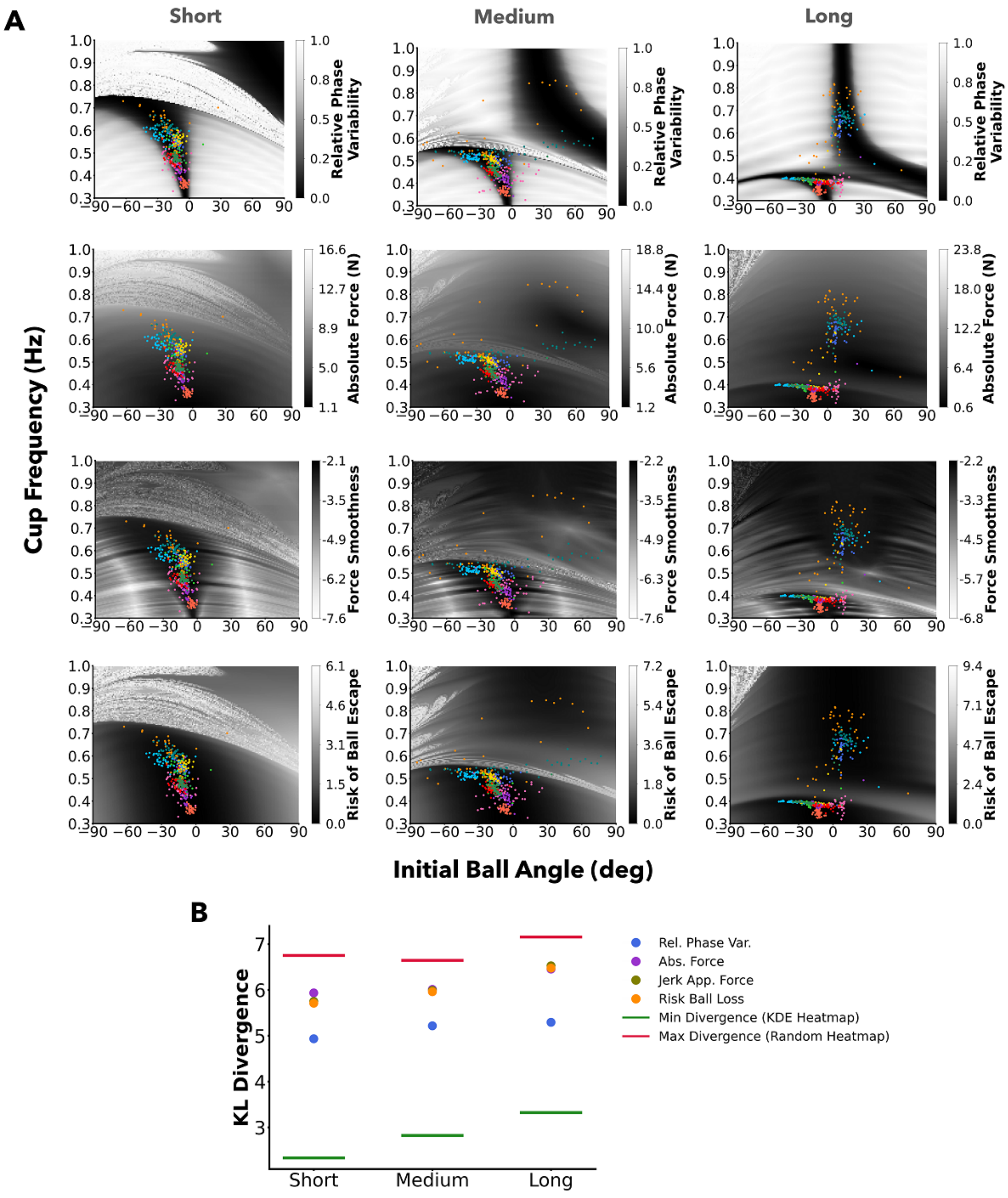

Fig A. Heatmaps for all variables and KL-divergence values for the random protocol. A) Heatmaps of the relative phase variability (row 1), absolute force (row 2), force smoothness (row 3) and risk of ball escape (row 4), for combinations of initial ball angles and cup frequencies plotted in grey shades in the background. Participant choices of initial ball angle and cup frequency from all trials are plotted as colored points in the foreground. Colors represent different participants. **B**) KL-divergence values between the

distribution of the participant data and the underlying heatmaps shown for the different variables and pendulums in **A**.

A heatmap of relative phase variability, absolute applied force, log dimensionless jerk of applied force, and risk of ball escape was plotted from simulation runs of all combinations of desired cup frequency and initial ball angle for each pendulum (Fig AA). Participant data from all trials was superimposed on the heatmap for the Random protocol.

The corresponding KL-divergence values between the participant data and the underlying heatmap are shown in (Fig AB). An estimate for minimum KL-divergence was obtained using a Kernel Density Estimate of the participant data as the underlying heatmap. An estimate for maximum KL-divergence was obtained using a random probability density heatmap as the underlying heatmap. The mean of KL-divergences from 100 randomly sampled probability density heatmaps was set as the maximum KL-divergence estimate.

Cost Function Descriptions for the Stochastic Optimal Open-loop Simulations

Considering the desired state trajectories and minimization of variance around the desired states, the following cost function was adopted:

$$J = (X_f - X_f^d)^T Q_f (X_f - X_f^d) + \mathrm{Tr}(Q_f^v P_f) + \int_0^{t_f} ((X_t - X_t^d)^T Q_t (X_t - X_t^d) + \mathrm{Tr}(Q_t^v P_t) + u_t^T R_t u_t) dt \quad (1)$$

where the subscripts t and f represent trial time and the end of trial (terminal) time, respectively. X and X^d are the state vector and desired state vectors respectively, P is the state covariance matrix, Q is the state cost weighting matrix, Q^v is the covariance cost weighting matrix. u is the control vector, R is the control cost weighting matrix, and Tr is the trace of the matrix. The total simulation time was $t_f = 8\ s$ with a step size of $\Delta t = 10\ ms$ resulting to a total of 800 simulation steps.

For reference, the state vector and the control vector were defined in the manuscript text as follows:

$$X_t = [x_t,\ v_t,\ \theta_t,\ \omega_t,\ l_t,\ F_t,\ K_t,\ \dot{F}_t,\ \dot{K}_t]$$
$$u_t = [\ddot{K}_t,\ \ddot{F}_{ff_t}] \tag{2}$$

Rhythmic Stage: The desired position trajectory of the cup and velocity of the cup was designed as $x^d = A\cos(2\pi ft)$ and $v^d = -2\pi fA\sin(2\pi ft)$, respectively. Quadratic costs were enabled on K and F_{ff} and their first derivative to limit true control magnitudes. The state weighting matrices Q_f, Q_f^v, Q_t, Q_t^v and the pseudo-control cost matrix R_t for the rhythmic stage ($time\ step : t \in (200, 800]$) of the simulations were designed as below.

$$Q_{t>200} = Q_{t=800} = 50diag(1,\ 0.1,\ 0,\ 0,\ 1^{-12},\ 1^{-12},\ 1^{-6},\ 1^{-6},\ 0)$$
$$Q^v_{t>200} = Q^v_{t=800} = \ 100diag(1,\ 0.5,\ 0,\ 0,\ 0,\ 0,\ 0,\ 0,\ 0) \tag{3}$$
$$R_{t>0} = 1^{-6}diag(1,1)$$

Preparation Stage: There were no desired cup or ball states during the preparation stage. Quadratic costs were enabled on K and F_{ff} and their first derivative. The state cost matrices for the preparation stage of the simulation ($time\ step : t \in [0, 200)$) were as follows:

$$Q_{t<200} = 50diag(0,\ 0,\ 0,\ 0,\ 1^{-12},\ 1^{-12},\ 1^{-6},\ 1^{-6},\ 0)$$
$$Q^v_{t<200} = 100diag(0,\ 0,\ 0,\ 0,\ 0,\ 0,\ 0,\ 0,\ 0) \tag{4}$$

Last Preparation Step: The desired state of the ball θ was varied in the simulation, and the state of the cup was $x = -0.15\ m$ and $v = 0\ m/s$. The state cost matrices for the last step of preparation stage ($time\ step : t = 200$) of the simulation were set accordingly.

$$Q_{t=200} = 50diag(0,\ 0,\ 10,\ 1,\ 1^{-12},\ 1^{-12},\ 1^{-6},\ 1^{-6},\ 0)$$
$$Q^v_{t=200} = 100diag(0,\ 0,\ 10,\ 5,\ 0,\ 0,\ 0,\ 0,\ 0) \tag{5}$$
