# Supplementary material for: Dynamical stability and mechanical impedance are optimized when manipulating uncertain dynamically complex objects
Source: PLoS Comput Biol. 2025 Dec 9;21(12):e1013797. doi: 10.1371/journal.pcbi.1013797 (PMC12704904; doi:10.1371/journal.pcbi.1013797)
Supplement: Attachment — Submitted filename: PLOS_C~1.PDF [file pcbi.1013797.s002.pdf]

October 2, 2025

Dear Dr. Berrett,

Thank you for the opportunity to revise and improve our work. We would also like to thank the reviewers for their comments. We have addressed each of their comments and suggestions in a point-by point-manner.

The reviewers' comments below are in bold text and our responses are in normal text format. Changes to the manuscript are in bold and blue (**bold and blue text**). The changes are reflected in the revised manuscript document in red. The figures in the responses and the manuscript are respectively referred to as **RevFig.** and **Fig.**

Sincerely,

Rakshith Lokesh and Dagmar Sternad

EDITOR COMMENTS

As you will see, two reviewers had minor concerns but one reviewer raised more major concerns. To address these issues, your revised manuscript should provide substantial clarification on the following points: (1) the novelty and rationale of this work compared to prior studies using a similar paradigm, (2) the hypothesis-driven approach that underlies the study, and (3) some key choices of the modeling and how it relates to the behavioral task. Please note that all relevant data should also be shared, if possible, according to the policies of the journal.

We thank the editor for providing an opportunity to address the raised concerns. We have mostly responded to the editor's major comments also in the general response to reviewer 3's comments. We summarize those responses in short here:

1. We continue to argue that our research topic is understudied in computational motor control compared to other research topics. While the work presented here continues our investigation into complex object control, it unifies and extends prior work by scrutinizing the effect of dynamical uncertainty and mechanical impedance in more detail.
2. We formulated all the hypotheses prior to analyzing the data. However, Hypothesis 3 was more a prediction following Hypothesis 2. Thus, we have refined the hypotheses with predictions and also provided more justification for our hypotheses.
3. We explained our choice of modeling uncertainty by referring to feedforward planning of impedance and elaborated how it relates to the experimental task.
4. We have also made the data available in the following public repository. OSF Data Storage.

After restructuring the order of methods and results, the example trial showing relative phase and relative phase variability calculations has been added as a separate figure (Fig. 2) in the Methods section. We also reordered the presentation of findings within the results section to match the flow of hypotheses. The cup frequency and initial ball angle trend results have been moved after the relative phase variability (Fig. 3) and preparation stage analysis (Fig. 4) results.

REVIEWER 1 COMMENTS

This study used a pendulum cart haptic system to investigate how human participants control an object with an internal degree of freedom. By looking at the cup's movement frequency and the ball's initial angle during the preparation stage, they examined how people change their behavior in the preparation stage depending on the uncertainty in the pendulum's length, which changed its dynamics. They found that participants' preparation behavior was best explained by a model that minimized the variability in the relative phase between the cup and the ball, i.e., by prioritizing predictability in

the task dynamics. The Introduction is thorough and appropriate, the Results are well described, the simulations are informative, and the Discussion and Conclusion reflect the study's findings. I have only minor suggestions and points that require clarification.

According to line 187 "the rhythmic stage started when the cup was first moved towards the box on the right side". But looking at Fig. 1C, it seems like the trial starts when it hits the left box. Is there a mistake in the text? Also, please state explicitly how the initial ball angle was calculated. Was it the angle when the cup first entered the box? Or was it its peak angle when inside the box on the first occasion?

We thank the reviewer for catching this mistake. For data analysis, the start of the rhythmic stage was identified as the first time that the cup velocity was zero before hitting the box on the right side. This time marks the transition from preparation to the rhythmic stage. Hence, the initial ball angle was also measured at this instance. We changed the text in the Methods to clarify these definitions.

Lines 251-254, Methods: **This time point, defined as zero time, represented preparation of cup-ball states before moving into the rhythmic stage. This time instant is different from the experimental start of rhythmic stage when participants first moved the cup into the right box.**

I would use the same color scheme in Fig. 3B as in Fig. 2.

We initially had the same color scheme for participants in Fig. 3B. However, the colors were overwhelming and could potentially be confused with other colors in Fig. 3A and C. Also, our research questions do not focus on inter-individual differences or individual participant's behavior. We therefore prefer to keep all the lines grey in Fig. 3B.

In Fig. 3C, please make the line styles different for each pendulum length and make the lines thicker. I found it difficult to discern which plot corresponded to which pendulum length.

We enhanced the color scheme, line thickness, and transparency of the error ribbons in Fig. 3C to make the lines distinguishable from one another.

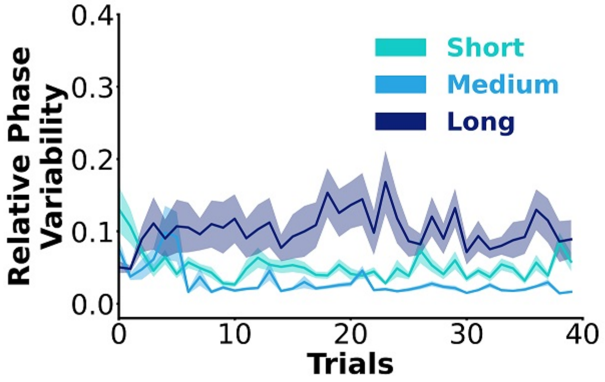

RevFig. 1 Updated color scheme and line width for Fig. 3C in the manuscript.

The authors used a three-way ANOVA to test the difference in the relative phase variability between the random and blocked protocols, but upon examining Fig. 3D, I don't think the data is normally distributed to warrant an ANOVA. Did the authors check that normality was not violated? Perhaps they could try taking a log of the relative phase variability and run their tests again to see if normality is preserved for further analysis with an ANOVA.

We thank the reviewer for raising this concern. Generally, ANOVA's are not sensitive when the dependent variables have moderate deviations from normality. However, the Q-Q plot as well as using the Shapiro-Wilk test showed that the distribution of relative phase variability deviated significantly from the normality assumption ($p < 0.05$ for all protocol and pendulum combinations). As the reviewer recommended, we used the log of the relative phase variability and ran our tests again. The normality of data was verified using Shapiro-Wilk test in R ($p > 0.05$, for all protocol and pendulum combinations). The QQ plots are shown below.

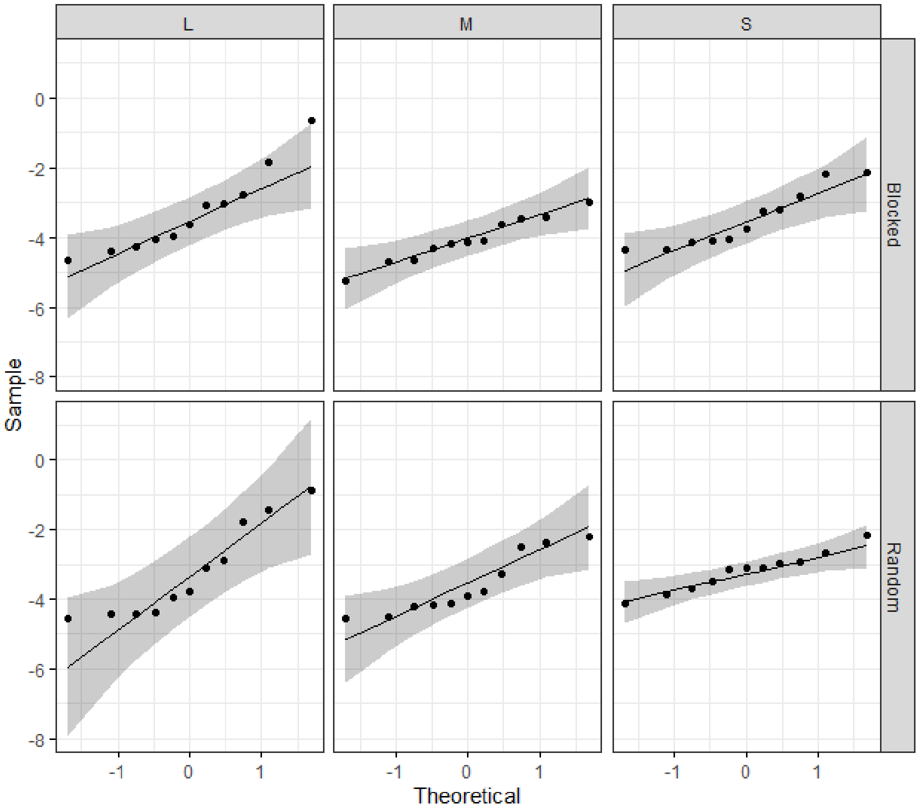

RevFig. 2 QQ Plot with logarithm of relative phase variability ensures normality.

Importantly, the statistical results and their interpretation did not change: there was no significant effect of pendulum length or protocol or their interaction ($p > 0.05$). The description of statistical analysis in the Methods and the statistical values in the Results section are updated.

Lines 444-446, Methods: **Prior to specific tests, normality of the dependent variables was**

checked using the Shapiro-Wilk test. In case normality was violated, the logarithm of the respective variable was calculated.

Lines 470-473, Results: **There was no significant effect of pendulum length on relative phase variability** $(F(2, 20) = 2.64, p = 0.095)$**. Relative phase variability (log-transformed) was not significantly different between the random and blocked protocol,** $(F(1, 10) = 3.90,\ p = 0.076)$ **(Fig. 3C).**

For Fig. 4B, were no post-hoc tests conducted? I would define the time taken to stabilize the relative phase when it is first introduced in the Results on line 283 instead of only in the Methods. Could the authors justify their selection of the 0.1 threshold? How was this determined?

There was no significant interaction effect between pendulum length and protocol for any of the three variables cycle number, preparation duration, or time to stable relative phase. Thus, post-hoc tests were not warranted.

We restructured the manuscript so that the methods section comes before the results section. Now time taken to stabilize relative phase is defined earlier.

The threshold of 0.1 was set by observing the low relative phase variability regions (darker regions) in the simulations (Fig. 5C). Thus, a relative phase variability of 0.1 sufficiently represented stable relative phase. We explained this choice of 0.1 threshold in the Methods.

Lines 286-288, Methods: **The threshold was selected based on the relative phase variability heatmaps from simulations in Fig. 6C. A threshold of 0.1 indicated stable (darker) regions for all pendulums.**

Concerning Fig. 4B, the number of cycles and preparation duration appear redundant, I would take just the latter since this is the most intuitive. Preparation duration and time to stabilize relative phase are different, but there is no further analysis beyond the three-way ANOVA. In the random protocol, the time to stabilize with the medium pendulum appears to be somewhat shorter than with a short and long pendulum, is this difference significant? Please add more details here, or if the conclusion is relatively the same, then I would replace all three metrics with just preparation duration.

We agree with the reviewer that number of cycles and preparation duration are redundant. However, it is not necessary that the preparation duration results should necessarily coincide with time to stabilize relative phase. Thus, we decided to leave out number of cycles and keep preparation duration and time to stabilize relative phase. We have updated Fig. 4B.

As we explain in the response to the previous comment, we could not perform post-hoc comparisons within the levels of Pendulum Length for the random protocol, as there was no significant main effect of pendulum length, nor an interaction effect. However, out of curiosity, we conducted pairwise t-test's between the pendulum conditions for the random protocol. They did not reveal significant differences for any of the pairs.

Line 329: "loosing the ball" -> "losing the ball"

We corrected this.

Is the plot in Fig. 5D available for all conditions? I didn't see Supplementary Materials showing these figures. I would add them either in the main manuscript or as Supplementary.

We left them out in the manuscript because the figure was overcrowded. However, we have included the plots for all conditions in the Supplementary Materials document.

Was the Kullback-Leibler divergence computed for each condition and then averaged across all conditions? Please specify.

We computed the Kullback-Leibler divergence for each pendulum (short, medium or long) and condition (blocked, random). We reported values only for the Random protocol in the text for brevity. We have specified the computations of the KL-divergence in more detail in the text as follows.

Lines 353-356, Methods: **For each variable, we used the Kullback-Leibler divergence to test how well the simulated heatmaps explained participants' choices of cup frequency and initial ball angle. We computed KL-divergence between the probability density of the heatmap and the 2D histogram of participant data for each pendulum and protocol.**

Lines 580-583, Results: **For brevity, we show heatmaps and report Kullback-Leibler divergences only for the random protocol and medium pendulum condition. The heatmaps and the corresponding KL-divergence results for other pendulum conditions are shown in the Supplementary document.**

There are several black splotches in the simulations of Fig. 7D, why did the stiffness produced by the controller sometimes drop close to zero in some cases, e.g., medium pendulum with cup frequency around 0.7 Hz and initial ball angle around 20 degrees?

The numerical optimization sometimes converged to very low stiffness values or stiffness profiles that dropped to zero. For example, for cup frequency (0.725 Hz) and initial ball angle (0 deg), the stiffness value dropped to 0 towards the end (Fig. 6). The underlying issue is a trade-off between minimizing stiffness and the variance of the states. Lowering variance requires higher stiffness, but higher stiffness results in greater control cost. It was better to lower stiffness than lower variance to reduce the total cost in Eq. 9. To overcome this issue, we increased the noise on pendulum length to $\sigma_l = 0.1$ for the blocked protocol simulations. This removed the black splotches and the heatmaps are more continuous. We updated the heatmaps and summary plots in Fig. 7D and Fig. 7B-C, respectively.

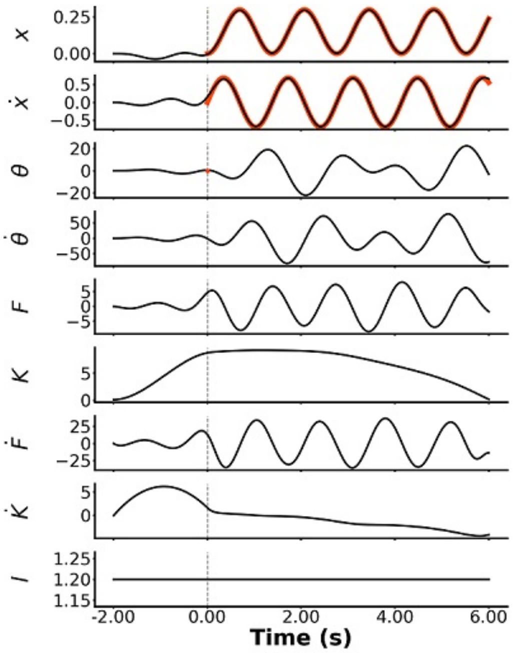

RevFig. 3 Numerical optimization converges to low average stiffness value.

I would recommend adding another subfigure in Fig. 7 like 7D but with the mean applied force just to see how stable it is for different conditions.

The force magnitude plots are qualitatively similar to the absolute force magnitude plot obtained using forward simulations in Fig. 5D. We have now included the recommended plots in Fig. 7E. We have also updated the Results and Figure caption as follows.

Lines 656-658, Results: **Stiffness (Fig. 8D) and absolute feedforward force (Fig. 8E) averaged during the rhythmic phase were plotted as a function of initial ball angle and cup frequency for the simulated blocked and random protocols.**

Lines 652-654, Fig. 8 Caption: **Model predicted average absolute feedforward force (F_{ff}) for various combinations of initial ball angle and cup frequency plotted in the background. There are some regions of overlap between low average absolute forces and low stiffness.**

The local fluctuations in grip force (which weren't modelled) could be an artefact of the coupling between grip and load (applied) forces and may not be indicative of a voluntary impedance control strategy (Hadjiosif and Smith 2015). This could be added to the Discussion.

We agree with the reviewer that grip force can be modulated in response to changes in reaction force on the hand from the cup. For example, grip force would be greater near the targets when slowing down the cup. Besides, the grip force can also be modulated based on the magnitude of the ball force that is fed back to the hand. We have now discussed such a coupling between

grip force and load force in the Discussion.

Lines 779-784, Discussion: **One potential reason for these variations could be the coupling between load force and grip force (Johannson and Westling, 1984; Hadjiosif and Smith, 2105), i.e., the impedance may be increased in response to an increase in the reaction force on the hand from the cup. The increase in impedance corresponded with an increase in amplitude of the applied force during the preparation stage.**

While the relative phase variability matches best with the experimental data, this does not preclude the possibility of a weighted combination of the metrics, e.g., relative phase variability and absolute force. I would add a sentence in the Discussion describing this limitation.

We agree with the reviewer that other measures could also be co-optimized along with relative phase variability. In fact, we discuss on Line 571 that maximizing stability also overlaps with minimizing absolute effort or maximizing smoothness of applied force. However, we did not further characterize the overlapping regions between the variables and whether participants converged onto these overlapping regions. We have discussed this possibility in the Discussion now.

Lines 738-742, Discussion: **There was indeed some overlap between maximizing stability and minimizing absolute effort or maximizing smoothness of applied force. It is therefore possible that humans optimize a weighted combination of the different variables. Further analysis is necessary to determine how maximizing stability could also lead to minimization of applied force.**

REVIEWER 2 COMMENTS

This article investigated an interesting question: what are the human motor control strategies during the dynamic manipulation of complex and uncertain objects. They used a simplified cart-pendulum model to simulate a nonlinear and under-actuated physical system, introducing system uncertainty by varying the length of the pendulum ball. The study found that humans adopt different preparatory strategies under higher uncertainty and prioritize dynamic stability for their motor control, increasing muscle stiffness to cope with uncertain dynamics. Feedforward simulations using an impedance controller and stochastic open-loop optimal control support these findings. The paper was interesting and enjoyable to read. I have the following comments to improve the paper and clarify some statements.

Major comments:

1. At Line 23 of Abstract, it states that “further revealing that participants selected preparation and interaction frequencies to minimize mechanical impedance”. It infers that the main goal for the motor control is to minimize mechanical impedance that is

related to the effort level, but previously from Hypothesis 3 it states that the dynamics stability was prioritized. Please make sure that the statements are consistent.

We thank the reviewer for raising this concern. There are two measures of effort here: i) Effort quantified as absolute magnitude of force applied on the object, ii) Effort required to grip the handle which is proportional to mechanical impedance of the arm. Thus, we specifically use 'magnitude of force' instead of 'effort' wherever applicable.

Besides, we do not want to suggest that stability is prioritized over other variables like mechanical impedance. Rather, a measure of stability best explains participant behavioral choice of preparation and interaction. It does not preclude the fact that choosing stable solutions also optimizes mechanical impedance or applied force magnitude, etc. as we explain in response to Reviewer 1's comments. We discuss on Line 571 that maximizing stability also overlaps with minimizing absolute effort or maximizing smoothness of applied force. We have discussed these possibilities in the Discussion section Lines 738-745. We have corrected the text in Abstract, Results, and Discussion to accurately reflect these ideas.

Lines 19-20, Abstract: **dynamic stability matched participant behavior better than magnitude or smoothness of force.**

Lines 21-24, Abstract: **Feedforward simulations using an impedance controller and stochastic open-loop optimal control corroborated these findings, further revealing that participants' selection of preparation and interaction frequencies also minimized mechanical impedance.**

Lines 579-584, Results: **Hence, we concluded that the choice of cup frequencies and initial ball angle of the participants was best explained as minimizing relative phase variability.**

Lines 661-663, Results: **Overall, these simulation results and the empirical data together suggest minimizing end-point impedance as a plausible explanation for participant's preparation and interaction strategies.**

2. At Lines 359-360, it states that "The average grip force, shown by the bold lines, gradually increased during the preparation stage." This is interesting as the dynamics are unchanged within the trial. The increase of the impedance is probably due to different requirements in the rhythmic stage. The applied force seems also having an increase in rhythmic stage compared to preparation stage from Fig. 6B. Please add discussions on the different behaviours between the two stages and potential reasons.

The magnitude of applied force increased in the rhythmic stage because the participants increased the amplitude of the cup going from the preparation stage to the rhythmic stage (as observed in Fig. 1C). It has been shown previously that the grip force is proportional to the magnitude of the load (applied) force (Johannson and Westling, 2015). Similarly, in our experiment, it is likely that participants increased the grip force to account for increasing inertial forces of the cup and the ball forces as the ball starts to oscillate more. We have discussed this

as follows in the discussion section.

Lines 779-784, Discussion: **One potential reason for these variations could be the coupling between load force and grip force (Johannson and Westling, 1984; Hadjiosif and Smith, 2015), i.e., the impedance can be increased in response to increase in the reaction force on the hand from the cup. This is consistent with the observation that increase in impedance corresponded to an increase in amplitude of the applied force during the preparation stage.**

3. In Fig. 3C, the medium and long colours are close, which are not very easy to distinguish. Besides, please describe Fig. 3C that the meaning of the solid line in figure caption. The long pendulum results (dark blue line) look consistently higher than the other two, however there is no significance detected from statistics. The results in Figs. 3C& D look not very consistent, please add some additional descriptions to clarify this. Besides, from 3B, it looks also that the long pendulum has more variance compared to the short and medium, and more switches between the 0 deg and 180 deg, especially for the random protocol. How would this reflect the task difficulty level or influences stability control?

We now use a different set of colors for the three pendulums as shown below for the random block.

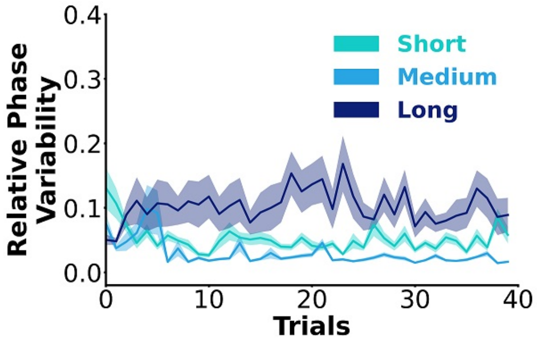

RevFig. 4 Updated color scheme and line width for Fig. 3C in the manuscript.

We also now described the solid line as the mean across subjects and the error ribbons as ± 1 standard error in the figure caption.

Lines 482-483, Fig. 3 Caption: **Mean relative phase variability across subjects plotted against trials for the three pendulum conditions in the two protocols. The error ribbons represent ± 1 standard error.**

We verified that the results from Fig. 3C and 3D are consistent with one another. The lines in 3C are averages across subjects, whereas the points in 3D are averages across trials for the same dataset. To clarify, relative phase variability was computed as the circular variance of relative phase within each trial. It does not capture variability between the trials or between subjects that is plotted in Fig. 3B.

However, it is interesting that average relative phase is more variable between the trials

for the long pendulum. This is primarily because the long pendulum ensures stability for both in-phase and anti-phase solutions (two branches in Fig. 5C long pendulum) in the frequency range of 0.3 to 0.7 Hz, while short and medium do not. Frequencies higher than 0.7 Hz were not preferred because of the larger force requirement. Thus, participants could flexibly adopt in-phase or anti-phase solutions with longer pendulum. We further discuss the flexibility in choice of frequencies as follows.

Lines 729-732, Discussion: **These areas afforded two disjunct areas that accommodated the in-phase and anti-phase solutions that subjects chose. Thus, a few participants also switched between the in-phase and anti-phase solutions with the long pendulum.**

4. In eq. (5), the double derivative of impedance and force are chosen as control input, however as human impedance modulation may relatively slow dynamics, controlling the double derivative of the impedance may impose constraints to the control inputs. This would turn it into a constrained optimal control problem. Please clarify whether these control constraints have been considered in the current modelling framework. Besides, could you please share some information on how using the (K,F) or their first derivative as control input would change the results?

The double derivatives of control were used as pseudo-controls mainly to ensure smooth changes in stiffness and force, and to initialize the force at 0 at the beginning of each trial simulation. Thus, we levied costs on the first and second derivative of the force (F_{ff}) and impedance (k) terms. We did not impose any other constraints on the control inputs.

When we optimize k and F_{ff} directly, we obtain the system states for a cup frequency $0.7Hz$ and initial ball angle of 0 deg as shown in RevFig. 5. The control variables, feedforward force and stiffness, do not change smoothly with time. We also generated the impedance plots as a function of initial ball angle and cup frequency. These are qualitatively similar to plots obtained by optimizing with double derivatives of the control variables as pseudo-controls.

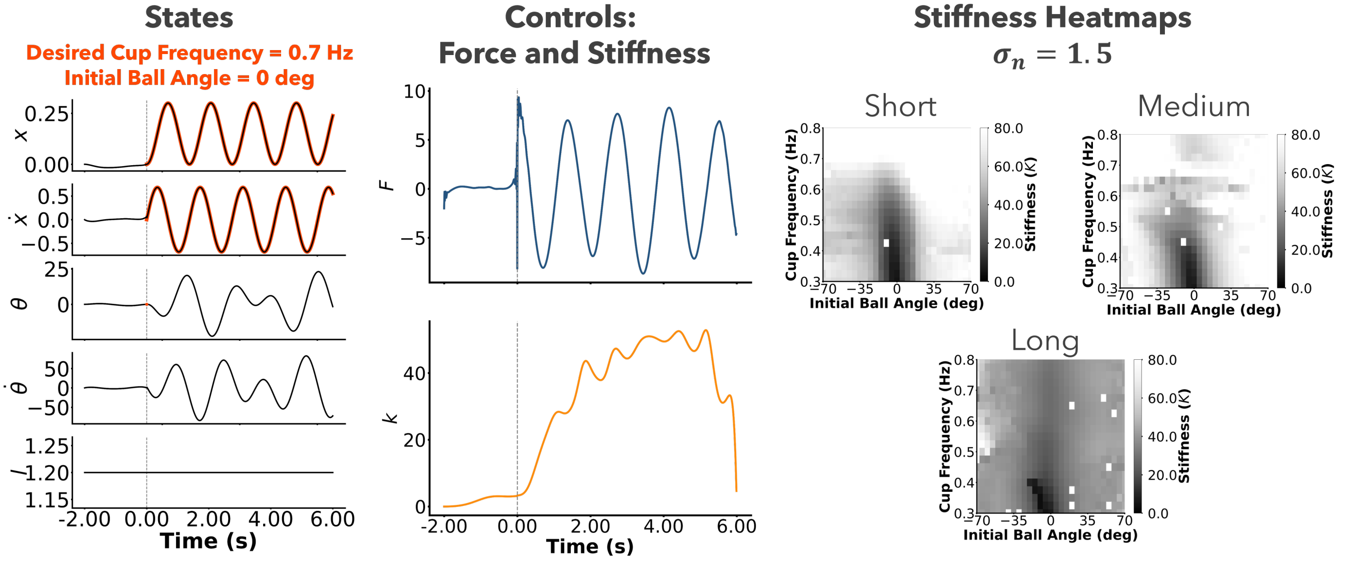

RevFig. 5 Stochastic Optimal Open-loop Control simulations by optimizing k and F_{ff} directly.

5. At Lines 915-916, "An initial guess for F was obtained using the impedance controller defined in Eq. 10 using a small gain K to improve the convergence speed of the numerical optimization." The F here is the feedforward force control, while the force in eq. 10 is more for feedback force control. Besides, eq. (10) and eq. (12) share the same symbol F but they have different meanings, please use different symbols for these two, otherwise there will be two feedback control terms in eq. (12).

To remove this potential source of confusion, we have renamed F to $F^{applied}$ in Eq. 10 consistent with Eq. 12. We renamed F in Eq. 12 to F_{ff} to indicate that it is a feedforward force term.

6. At Lines 574-575, the authors claim that humans prioritize objectives going beyond effort and smoothness, however I am entirely convinced by this claim. As they mentioned that people fatigue may change the results, I was wondering whether authors have looked at how the gripping force changes along trials and whether the Kullback-Leibler divergence values are consistent between the beginning and finishing trials. From Fig. 3C, there is a slight increase trend in the short and medium pendulum between trials 30 and 40. Some further analysis would be necessary to support this claim.

We might expect the grip force to drop with a larger number of trials due to two reasons: i) participants learn to stabilize the dynamics without the need for high impedance, ii) fatigue. However, in our experiment we did not observe any changes in the magnitude of grip force with trials (Fig. 5). This can be seen from the complete KL-divergence results.

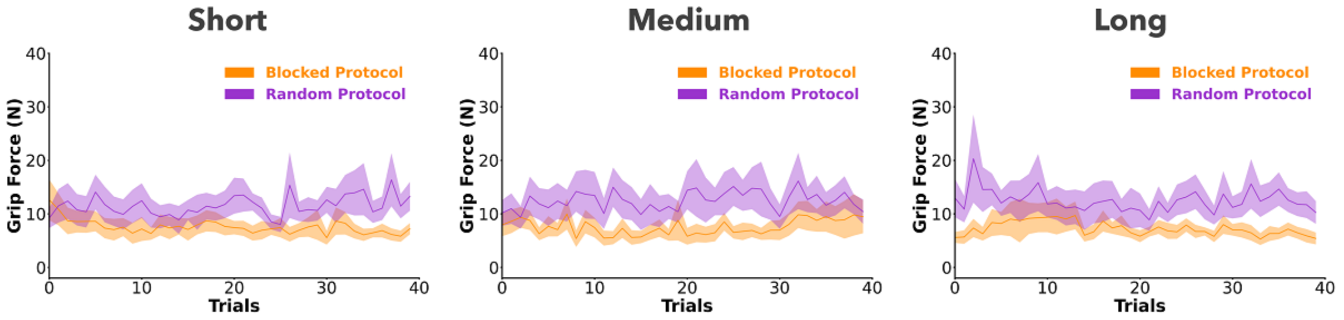

RevFig. 6 Average grip force from all participants did not change as a function of trials.

The KL-divergence for all trials, first 10 trials and last 10 trials, are shown in the (RevFig. 7) below. Minimum and maximum estimates for KL-divergence for the given experimental data are also indicated in the plot for reference. The minimum estimate was obtained by assuming a Kernel Density Estimate of the data as the heatmap. The maximum estimate was obtained by assuming a randomly distributed underlying heatmap and computing the mean KL-divergence from 100 random samples. The KL-divergence values did not vary much between the first 10 and the last 10 trials. As mentioned in an earlier comment, we think that the KL-divergence values sufficiently support our claim that stability explains participant choices of preparation and interaction better than absolute force, or smoothness.

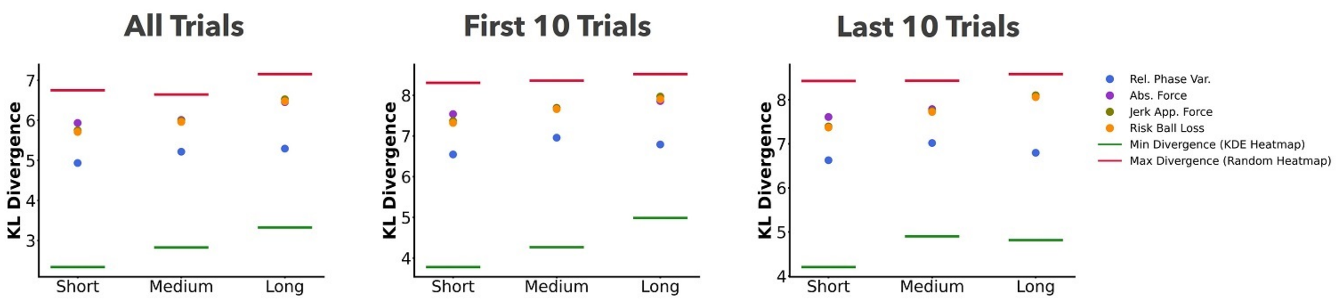

RevFig. 7 KL Divergence values for all pendulum conditions and optimization variables.

Besides, the authors mainly consider the force smoothness in Fig. 5D, but the movement smoothness is important to consider, so it would be beneficial to analyse the movement smoothness.

The hand/cup movement smoothness is essentially determined by the desired cup trajectory used for the simulations, i.e., $x^d = -ACos(2\pi ft)$. Thus, the cup movement smoothness would be moderately impacted, only based on the desired frequency (f). On the other hand, the force profiles can change drastically for the same desired cup trajectory, depending on the initial condition of the ball and the subsequent dynamics of the ball (Nasseroleslami et al., 2014). The intricate force smoothness heatmaps in Fig. 5D and the Supplementary document (Fig. 1A) reinforce this choice.

Minor:
1. At Line 195, it told that there are "210 trials" in total for each protocol, however Fig. 1D shows that there are "120 trials", please make sure that the number of trials is correctly reported. Similar issue exists at Line 196.

This was a typing mistake. The number of trials was 40 per pendulum per protocol. We have updated as follows.

Lines 231-234, Methods: **Two experimental protocols (Fig. 1D) were tested: random protocol - the pendulum length was varied randomly trial to trial (120 trials in total), and blocked protocol - the pendulum length remained constant within a block of trials (120 trials in total).**

2. At Lines 237-238, anti-phase relations are mentioned without further explanations. Please give some descriptions on whether anti-phase relations are good or bad to the stability.

The anti-phase relation permits similar degrees of stability as that of in-phase relation as evident from the heatmaps shown in Fig. 5C. Here, the two branches at the top right and the bottom left correspond, respectively, to anti-phase and in-phase relations. Further analyses and experiments would be required to determine which branch is better for stability, i.e., has a greater area and is more robust to sensorimotor noise or perturbations. We have explained the details

in the Results as follows.

Lines 543-548, Results: **The medium and long pendulums supported both in-phase and anti-phase solutions (two dark colored branches) in the preferred frequency range of $0.3\ Hz$ to $0.7\ Hz$ of the participants. Some participants adopted both in-phase and anti-phase stable solutions indicated by the same colored dots in the in-phase and the anti-phase branches, as well as evident from the crossing over of lines from $0\ deg$ to $180\ deg$ in the relative phase plots in Fig. 3A.**

3. In Fig. 5D, the force smoothness is flipped for some reasons, but it would be better to make it consistent with others.

We defined the grayscale scheme such that darker regions indicate better solutions, irrespective of the variable and its sign. This convention allows readers to visually compare the heatmap to the overlaid data.

4. At Lines 423 & 878, the dimension of u should be 2 instead of 3.

Thank you. We corrected the dimension to 2.

5. At Line 914, B is not used in the formulation. As the control inputs are the double derivative, should these initial guesses be the double derivative of K and F correspondingly?

We generated an initial guess for the second derivative of the feedforward force term. The initial guess for $\ddot{K}$ was set to zero. The initial value for K was set to 10 and $\dot{K}$ was set to 0. We corrected the description as follows:

Lines 434-436, Methods: **The initial guess for the pseudo-control $\ddot{K}$ was set to zero for all t. The initial guess for K and $\dot{K}$ at $t = 0$ were set to 10 and 0 respectively to prevent divergence to negative values.**

6. In eq. (9), the lower bound of the integral should be added.

We added the lower bound of the integral.

7. In eq. (8), please define clearly what is the θ_{rim}.

The value of $\theta_{rim} = 60\ deg$ has been specified as follows.

Lines 345-346, Methods: **The escape energy was the maximum possible total energy of the ball before it escaped beyond the rim of the cup ($\theta_{rim} = 60\ deg$)**

8. At Line 749-750, it states that "In the preparation stage, participants were encouraged to explore the cup and ball dynamics by 'jiggling' the cup between the left block

(−0.15 m) and the midpoint at 0 m.", However, in Fig. 1B, the midpoint position is not provided in the visual feedback, how does the participant know the midpoint position? Besides, why space between left block and the midpoint is selected in the preparation stage? Would this influence the results of preparation if subjects have different handedness?

The instruction was to oscillate the cup anywhere on the left side of the right block at $+0.15\ m$. The participants were told that the rhythmic stage began as soon as the cup was moved into the right side box for the first time. We centered the participants such that their shoulder was in line with the midpoint of the two blocks. We do not believe handedness would influence preparation, since they could use all the space on the left side of the right green box. We have updated the text as follows:

Lines 219-222, Methods: **In the preparation stage, similar to prior experiment (Nayeem et al., 2021), participants were encouraged to explore the cup and ball dynamics by 'jiggling' the cup anywhere on the left side of the right green box, Box B ($0.15\ m$).**

9. At Lines 751-753, it states that "When participants were ready to start the rhythmic stage, they moved the cup towards Box B, and then continuing with rhythmic back and forth movements between the two target boxes." Please add more specifically what information has been provided to the participants: are they aware of the trial duration or whether they are informed of any rewards or penalties on the movement control? This would be useful to understand their motion and impedance behaviour.

The participants were informed that they had to oscillate the cup for 15 seconds. They did not receive any feedback in terms of rewards or penalties on their task performance. The only instruction was to maintain a consistent rhythm and to keep the ball within the cup during the rhythmic stage. We have added details about the instruction given to the participants as follows.

Lines 225-227, Methods: **For the rhythmic stage, participants were instructed to oscillate the cup with a consistent frequency of their choice without losing the ball for the next 15 seconds.**

10. At Line 706, please add the mean and standard deviation of participants' age.

We added the mean and standard deviation age.

11. At Lines 736-737, "an admittance-controlled robotic manipulandum (HapticMaster, Motekforce, Netherlands, Fig. 1B)." Is the admittance-control used in the experiment? The admittance-control may changes the dynamics of the system. If I understand correctly, the dynamics eq. (6) was implemented in the handle movement, please clarify this.

The HapticMaster controls position of the end-effector (handle) using admittance-control which is a low-level controller. The controller acted to faithfully reproduce the position of the end-

effector determined by the model of the cup-ball system. We did not use admittance control in any aspect related to our research.

12. In Fig. 7D, the simulation results in the low stiffness area of the long pendulum blocked plot seems not very continuous compared to short and medium plots, can you please give some explanations for that?

Reviewer 1 also pointed out the low stiffness areas in these plots. In short, the numerical optimization sometimes converged to very low stiffness values or stiffness profiles that drop back to zero. For example, for cup frequency (0.725 Hz) and initial ball angle (0 deg), the stiffness value dropped to 0 towards the end (RevFig. 8). The underlying issue is a trade-off between minimizing stiffness and the variance of the states. Lowering variance requires higher stiffness, but higher stiffness results in greater control cost. It was more optimal to lower stiffness than lower variance to reduce the total cost in Eq. 9. To overcome this issue, we increased the noise on pendulum length to $\sigma_l = 0.1$ for the blocked protocol simulations. We updated the heatmaps in Fig 7D.

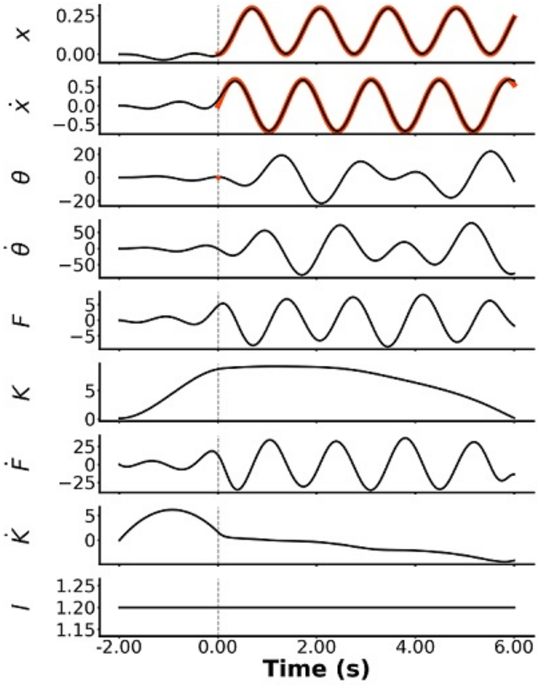

RevFig. 8 Numerical optimization converges to low average stiffness value.

13. At Lines 489-491, "Stiffness and applied force... simulated random protocol (Fig. 7D)" However, only stiffness has been displayed in Fig. 7D.

Thank you. We added the applied force plots in Fig. 7E and updated the figure caption.

14. For the cost function between Lines 437 and 438, what are the desired values used for the stiffness and force as well as their derivative in the desired states?

We added control costs on the second derivatives to ensure smooth changes. However, we still needed to impose costs on the magnitude of stiffness and feedforward force using the state cost weighting matrices. Thus, the desired states for stiffness and feedforward force were set to zero. Effectively, quadratic costs on the 'real' control variables , i.e., magnitude of force and stiffness, were imposed. We included the cost function matrices in the Supplementary document.

REVIEWER 3 COMMENTS

This paper, overall, presents several interesting and potentially important ideas. I have quite a few specific (line-by-line) comments below, but they generally can be lumped under 3 general categories, 1 fairly straight-forward, 1 likely manageable, but one potentially challenging:
On the straight-forward side, there is a lot of duplication across "Results" and "Methods". This is distracting and unnecessary. But it can also be relatively easily resolved by more clearly separating "Results" from "Methods" – in whichever order the authors feel it is best to present these.
"Manageable" is the conceptual issue of presenting this work as testing multiple "hypotheses" that are really not "hypotheses" but instead "predictions" and "explorations" of the experimental manipulations and results. Again, however, this should be somewhat easy to resolve in re-writing the narrative.
Most challenging is the question about the "So What?" question. It is very clear that a lot of work was done – experimentally, analytically, computationally, etc. however, it is not at all clear in the current narrative "To what end?" – i.e., what new important insights this work has to offer beyond a now extensive body of work already published (mostly, but not all, by this group) on this general ball-cup paradigm and these general questions surrounding "dynamic stability", "impedance" and "what do people control and how?" when performing these tasks.
The specific comments below articulate the reasons for this assessment and offer (I hope) a roadmap...

We thank the reviewer for the overview of comments. We start with a general response to the three main concerns and further address the individual comments below.

GENERAL RESPONSE:

1. *Regarding overlap between methods and results:* We thank the reviewer for the suggestion to change the sequence of Methods and Results. We therefore restructured the manuscript accordingly which removed redundant information and significantly reduced the length of the results section. After restructuring the order of methods and results, the example trial showing relative phase and relative phase variability calculations has been added as a separate figure (Fig. 2) in the Methods section.

2. *Regarding formulation of hypotheses:* We understand the reviewer's concern to some extent and reformulated the hypotheses to clarify their motivation and basis. However, we would like to remark that the distinction between hypothesis and prediction is far from being agreed upon in the community. Hypotheses can be more general theoretical expectations, based on first principles reasoning or they can be developed from previous results. They may be formulated in more general scientific terms or in operationalized form that directly affords statistical testing. We tended to use the latter. But critically scrutinizing our hypotheses and trying to integrate the reviewer's concerns. The following motivations are now integrated in the text:

   (a) *Hypothesis 1*: The preparation stage is important to stabilize transient dynamics when dynamic uncertainty is high. The underlying idea was that participants will need to explore more to find the initial conditions that help stabilize the system, especially under uncertainty. The statement that preparation times are longer and lead to gradual stabilization of relative phase are predictions based on Hypothesis 1.

   (b) *Hypothesis 2*: Humans covary preparation and cup frequency to maximize stability. This hypothesis was theoretically motivated because initial conditions and input frequency affect stability of a nonlinear system. Also, previous work dating back to Haken, Kelso and Bunz (1985) has shown that humans prefer stable movement solutions. The third hypothesis (Hypothesis 3) that humans prioritize stability over other variables was also inspired by analyses conducted by Nayeem et al. (2021). However, we do agree that it was more of a follow-up from Hypothesis 2, i.e., does stability explain human choice of preparation and cup frequency better than other commonly adopted variables like force magnitude and smoothness?

   (c) *Hypothesis 3 (Hypothesis 4 in the initial draft)*: Humans increase their arm impedance to account for uncertainty in dynamics. This hypothesis was justified given prior findings that humans increase end-point stiffness to control motor variability (Franklin et al., 2003; Gribble et al., 2003).

   We have retained Hypotheses 1, 2 and 4 with added justification for each hypothesis. We have rewritten Hypothesis 3, making clear that it contingent upon Hypothesis 2 being supported by the data. As stated, these hypotheses were formulated prior to the experiment.

3. *Regarding scientific contributions*:

   Human manipulation of underactuated and nonlinear objects has been explored over the past 2 decades. However, it has been a significantly understudied research area. There have been four primary research groups that have researched this area with a series of experiments and publications; Sternad/Hogan, Insperger/Milton, Franklin/Leib, and Mussa-Ivaldi/Dingwell. The number of relevant journal publications across these four groups is around 18 over the past 20 years. This is very little research in comparison to, for example, the amount of work published over the past two decades on reaching in the horizontal plane with a focus on adaptation. Moreover, the groups working on complex object control have focused on distinct research questions concerning sensory feedback, internal

representation of dynamics, mechanical impedance, and interaction strategies. Furthermore, they used a wide variety of approaches including dynamical systems analysis, control modeling, and open-loop simulations, etc. Besides, research in robotics has recently laid greater emphasis on enabling robotic manipulation of dynamically complex objects where human performance still serves as a benchmark (Zhu et al., 2022, IEEE Robotics and Automation Letters). Overall, we would consider research on human manipulation of complex dynamical objects to be significant and still understudied.

The first work from our lab in this series of publications was in 2014 (Nasseroleslami et al., 2014). Since, then there are 4 publications that have advanced insights on sensorimotor control of complex dynamical objects (Maurice et al., 2017; Bazzi and Sternad, 2020; Nayeem et al., 2021; Sharif Razavian et al., 2023), excluding conference submissions that have led to the journal publications. The work has focused separately on interaction frequency, control contraction metrics, preparation of object states, and mechanical impedance. Our present work clearly builds upon some of our earlier work. However, it significantly advances insights by: i) addressing a new research question, ii) tying the previous results on preparation and interaction together, and iii) expanding findings on mechanical impedance. Building upon prior research, while advancing new insights is the general requirement for any systematic research and also necessary for obtaining grant funding.

We mention the specific important findings from our work that goes beyond our prior work:

(a) Novel experimental design that tests the effect of uncertainty in dynamics on preparation, interaction and impedance strategies;

(b) Preparation and interaction are flexibly chosen, but in an interdependent fashion to ensure dynamical stability. Furthermore, we demonstrate with analytical measures that stability explains participants' control strategies better than other conventionally used cost measures like effort or smoothness.

(c) Capturing modulation of mechanical impedance (end-point stiffness) of the arm using a novel grip sensor.

(d) We apply the Stochastic Optimal Open-loop Control model to reproduce participant grip force characteristics arising in the experimental manipulation.

(e) Lastly, we are not aware of prior work that has predicted using computational modeling that preparation and interaction can be selectively chosen to also minimize the mechanical impedance.

While some of our results are intuitive, it is necessary to demonstrate them using carefully designed experiments supported by appropriate data analysis and computational modeling. Additionally, some of our findings open avenues for further research.

As the relevance of our findings seemed to not be evident to the reviewer, we now incorporated changes across the manuscript to sharpen the motivation and significance, and explicitly provide implications of our research.

ABSTRACT:

As currently written, it is not clear if the "hypotheses" came before the experiments and data... Or the experiments and data came first. As written, the experiments are described first. So it's not clear that these were "hypotheses" or merely "findings".

As explained in the general response, we updated the abstract text.

Lines 15-21, Abstract: **The results supported three hypotheses: 1) When dynamic uncertainty is high, object preparation is important to stabilize the transient dynamics; Stability increases during preparation and humans prepare longer when the dynamics is uncertain. 2) Humans maximize dynamic stability by flexibly covarying system preparation and cup frequency 3) Humans increase their arm impedance to accommodate uncertain dynamics, while the net force applied on the cup remains the same.**

LL. 23-25: The general overall conclusion does not appear substantively or substantially different than multiple prior papers by the senior author's group. The group has done very strong work over more than a decade now on this paradigm and the general question (L. 5) of "It remains unclear what motor control strategies ensure stability..." etc. The current abstract gives little-to-no indication what truly new, or novel, or important but as-yet-unanswered questions this present work offers.

Essentially, the more specific question is, how preparation, interaction frequency and mechanical impedance contribute to ensure stability. The brevity of the abstract makes it difficult to be more specific, but the formulation of hypotheses clearly communicates the research motivation. We developed the arguments in more detail in the Introduction section.

AUTHOR SUMMARY:
See comments above regarding the abstract. Same issues here. As written, it's a nice description of what was done and what the results were. But that is about all. Does not answer the "So What?" question.

INTRODUCTION:
LL. 46-65: A nice overview... But seems somewhat dated given the extended prior work on manipulating "dynamically complex" objects, by this group and others, over the past 2+ decades now.

We wish to revisit the earlier findings and motivate our work for the same reasons that this field is largely understudied. For readers, who have not read the few papers from our lab, this should build up to the specific research questions.

LL. 65-66: 50-52: The starting statement that "To date, only a few studies have explored..." is incommensurate with (1) the number of studies then described that have addressed this question, and the fact that those studies date back to at least... looks like

2002 is the oldest one cited here? So more than 2+ decades of work on this topic area now?

We addressed the concerns in our general response above.

LL. 89-90: Unclear. Sensitivity to initial conditions (i.e., 'the butterfly effect') is typically a hallmark of chaotic nonlinear systems... But many systems are nonlinear and not "chaotic". To my knowledge, the ball-cart system studied here, while certainly "nonlinear" is not "chaotic" – or at least is not studied in any chaotic region of its dynamics (even if such regions exist mathematically), as people in fact do maintain stability of their overall movements in these experiments, as do the model simulations.

The forced cart-pendulum system can exhibit chaotic behavior, especially when it is forced. The relative phase variability (a proxy for stability) is highly sensitive to cup frequency and initial ball angle. We observed this in the heatmaps shown in Fig. 5C and 5D. There are regions in the heatmap where small changes in initial ball angle discontinuously changes relative phase stability or absolute force, force smoothness or the risk of ball escape. Also, the stroboscopic plot borrowed from Nasseroleslami et al. (2014) highlights that the predictability of force is highly sensitive to the initial ball angle.

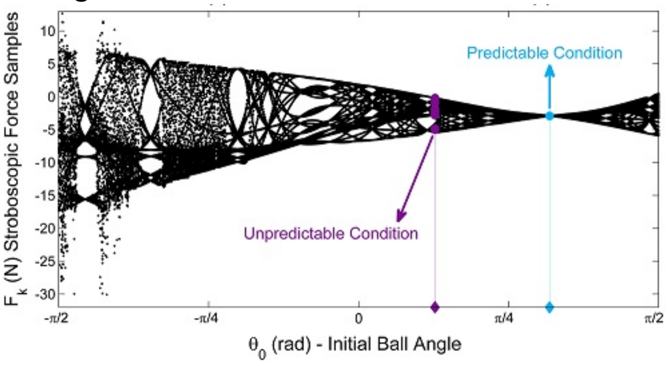

RevFig. 9 Force stroboscopic plot (Nasseroleslami et al., 2014) shows force predictability is sensitive to initial conditions.

LL. 100-101: While this paragraph (LL. 89-100) summarizes prior work from this group and while "it remains unclear..." But the Intro does not articulate how or why this question is important, beyond being the next incremental step in disentangling this specific paradigm that the senior author has been working on for (at least) 15+ years now.

Our prior work addresses different research questions about human manipulation of complex dynamical objects. The statement 'it remains unclear' refers to our attempt in unifying the findings from prior work and extending the findings to scenarios when the object dynamics is uncertain. Eventually, the finding that humans are sensitive to nonlinear dynamics and flexibly covary preparation and interaction strategies to ensure stability, especially when the dynamics is uncertain, has not been seen before, and is not an incremental finding. As mentioned above, systematic extension of insights into a complex problem is a widely accepted way of doing

research.

LL. 102-118: The concept of regulating mechanical impedance has been around 4+ decades now (Hogan, 1984 etc.). And the senior author has at least 4 prior papers on this ball-cup paradigm that incorporate at least some aspects of mechanical impedance in their models (Maurice, 2018; Nayeem, 2021; Razavian, 2023; & Bazzi, 2024). While this narrative summarizes key prior findings, it does not (by itself) provide a strong basis for the present study.

The previous papers did not incorporate dynamic uncertainties, nor did they measure impedance or allow variations in mechanical impedance in simulations. We mentioned these gaps in Lines 121-125 of the introduction.

LL. 110-111: "... plan the impedance in a feedback-based manner, eliminating the need for feedback-based corrections..." doesn't seem to make sense ("feedback" used twice). I suspect one of the "feedback" was intended to be "feed-forward"?

We corrected it to feed-forward.

LL. 118-122: Again (see comment on LL. 102-118) – The argument as presented that "These models did not allow... Therefore, it is crucial to explore..." does not articulate *why* "it is crucial to explore..." – especially given the extensive prior work on these ideas and even within this specific ball-cup paradigm.
LL. 126-127: The Intro describes how this new work builds on previous work from this group... But does not articulate how or why the new ideas and/or results presented here are more than an incremental improvement over that (now extensive) prior work.

The Lines 124-125 indicate that this work is an important step to understand how humans "modulate mechanical impedance" when interacting with complex dynamical objects. We already wrote in an earlier comment how this work goes beyond our prior work.

LL. 135-143: While "hypotheses" are presented, it is not at all clear where they came from or how they emerged from prior work. Furthermore (and perhaps more importantly), they are not so much "hypotheses" as (experimental) "predictions" – i.e., they predict the behavior the authors expected to observe in their experiments, but they do not address underlying physiological (or other) mechanisms that would (or might) be responsible for those observations.

We understand the reviewer's concern that the bases for our hypotheses were not sufficiently not provided. We have now justified each hypothesis as already stated above in the general comments.

Lines 138-148, Introduction: **We tested three main hypotheses: 1) Object preparation is important to stabilize dynamics when dynamic uncertainty is high, because more ex-**

ploration would be required to stabilize the dynamics when the system parameters are uncertain. Therefore, preparation duration will be longer in the random condition. 2) Humans maximize stability of dynamics by flexibly covarying the system's initial conditions and the following cup frequency, because initial conditions and interaction frequency affect stability of the system. Therefore, relative phase variability (stability) explains participant choice of preparation and interaction frequency better than absolute applied force or smoothness of applied force. 3) Humans increase their arm impedance but not net force to account for uncertain dynamics; arm impedance likely helps attenuate the variability in the system states.

RESULTS:
LL. 145-193: Most of this description is "Methods" not "Results". And most of it describes the same methods used in many prior papers on this paradigm by this group. It is not made at all clear which aspects of these tasks were 'same as used in prior studies' versus which were unique and new to this study.

The system equations, model parameters except pendulum length, task parameters, task instructions, and experimental equipment are the same as in Nayeem et al., 2021. We made clear what is the same and what is new in the methods section.

Lines 211-212, Methods: **The system equations, system parameters, task parameters, task instructions were the same as in the experiment by Nayeem, et al. (2021).**

LL. 189-191: It is not at all clear here how using different pendulum lengths manipulates "uncertainty in the cup-ball dynamics"... But this is a sequencing issue. The manipulating of uncertainty part is not about the different pendulum lengths themselves, but rather the random vs. blocked protocols... But those are not described until the next paragraph... (so just some gentle re-ordering of the description here I think...)

This problem has been resolved with restructuring of methods and results.

LL. 161-167, Fig. 1D: It seems the "Random Protocol" was conducted (for all participants) on Day 1, while the "Blocked Protocol" on Day 2.
LL. 204-205: However, the statement that "order... was counterbalanced" contradicts what is shown in Fig. 1D... Or Fig. 1D is not consistent with how the experiment was actually performed. One or the other or both should need to be fixed to clarify.

The order of random and blocked protocol was counterbalanced across participants. We added a line in the caption of Fig. 1D that the order of the two protocols was counterbalanced across participants. We also added bidirectional arrows to indicate counterbalancing in Fig. 1D.

Lines 193-194, Fig. 1D caption: **The order of random and blocked protocol was counterbalanced as indicated by the bidirectional arrows.**

Pgs. 4-7: In general, most all of what is presented on these 3½ pages is "Methods" not "Results". PLoS Comp Bio does allow authors to present their work as Intro-Methods-Results... If the authors feel this "Methods first" approach is best here, the manuscript should be restructured accordingly. If they believe the "Results first" approach is more suitable, then most all of this "Methods" description should be moved to "Methods" accordingly.

We thank the reviewer for the recommendation. We restructured the manuscript as described in the general response.

LL. 215-228: This is a lengthy description to end with the conclusion that there's "nothing to see here"...? It seems like 1 or 2 brief sentences would suffice, rather than two whole paragraphs.

The first paragraph is required to explain what the cup frequency and ball angle represent. The second paragraph is important because the unsystematic results are not obvious; it also builds up into the section where we show that preparation and interaction covary. However, the restructuring shortened the explanations.

LL. 229-261 & Fig. 3: It is not made clear if these results represent anything new or different from prior experiments using this same paradigm. If this and the prior section (Figs. 2 & 3) are more "quality check" () (or "confirmatory" – the paradigm 'worked'?) than new "Results" then consider moving to a Supplement and shorten the descriptions in the main paper to a sentence or two (e.g., "We confirmed that... (see Supplement ##)..." etc.)
LL. 262-286 & Fig. 4: Not clear that the prior comment might not also apply here as well....

This is the first time we tested random protocol versus blocked protocol. Hence, these results have not been presented in prior papers. These details and figures are required to comprehensively understand the experimental task, participant behavior, and the relative phase variability measure, especially for those readers who are not familiar with our work. Nevertheless, the results were shortened by the restructuring.

L. 263 vs. L. 285: More importantly, L. 263 states "Given these first seemingly unsystematic results, we determined..." So these analyses were conducted after obtaining and observing the experimental finding. Yet, L. 285 states "These results showed support for Hypothesis 1..." If both statements are true, this would seem to be a clear case of the (usually unethical) practice of "HARKing" (https://en.wikipedia.org/wiki/HARKing). Instead, I believe this was not a "test" of a "hypothesis" but instead a "prediction" based on preliminary observation of the date (as L. 263 indicates). The result itself is fine... But just should not be framed as testing a "Hypothesis" if that is not what was done.

Our statement "Given these first seemingly..." does not make sense in this context because analyzing the preparation stage does not resolve the "seemingly unsystematic results" in any way. If anything, the results under the next section on covariance between cup frequency and initial ball angle should resolve the seemingly unsystematic results. We corrected the sentence as below:

Lines 488-489, Results: **To address Hypothesis 1, we analyzed how relative phase changed during the preparation stage.**

LL. 288-291: Same problem. "Having seen..., we examined...." (LL. 288-290) clearly frames this as an exploratory (not apriori hypothesis-driven) analysis. That is contrary to "To test Hypothesis 2, ..." (LL. 290-291). Exploratory analyses are fine, and in fact often necessary, especially in computational work... But should be framed and presented as such.

Both statements were an attempt to generate a smooth narrative across sections. They do not reflect how our thinking proceeded and how we conducted the analysis. Our group performed similar forward simulations using an impedance controller before in Nayeem et al. (2021). Hence, we ran simulations for the different pendulums even before examining the participant data. We removed the transition statements.

LL. 291-301: Too much (and too detailed) "Methods" description for a "Results" section...

All these details have been moved to the Methods section.

LL. 327-347: Again, same issue as above – The simulations offer post-hoc insights into the already-obtained and observed experimental findings (which is fine!), but they do not test some a priori "Hyopthesis"...
This section, In General: The simulations themselves seem fine, but as presented, were clearly devised a posteriori - *after* the experimental results had been obtained and assessed in some sense. Such simulations help "explain" (a posteriori) and offer additional context to the experimental results obtained... But that is very different than testing some (a priori) "Hypothesis" (e.g., L. 324 & L. 327, etc.).

All these statements were removed with the restructuring. Also, the hypotheses were reformulated as described above.

Lines 583-584, Results: **These simulation results underscored the experimental results and supported Hypothesis 2.**

LL. 339-342: Numerically, yes, the K-L divergences for minimum relative phase are "smaller" than for the other criteria (shown in Fig. 5D). However, (1) Fig. 5D shows the experimental data lying also at relatively (close to?) minimal values of those alternative criteria

[abs. force, force smoothness, risk of escape], and (2) context for the numerical K-L divergence values is missing: i.e., what does a K-L divergence of 4.93 "mean" – physically and/or functionally etc.? How physically and/or functionally meaningful are the differences between, say, 4.93, 5.93, 5.74, and 5.70 (taking the "short" values just as examples)? Though the minimizing relative phase variability criteria seems "slightly better" than the others tested here, based on this K-L divergence metric, the data themselves (Fig. 5D) don't seem to unequivocally rule out the other criteria, or some combination of these various criteria. This is a long-standing and somewhat common issue in motor control (e.g., https://doi.org/10.1006/jmps.2000.1295).

We appreciate the comment. We do not claim that humans exclusively minimize relative phase variability. As the reviewer also points out, there are overlapping regions between relative phase variability and the other metrics. It is highly likely that humans minimize effort and smoothness of force in our task. However, it is useful to have a single variable, i.e., relative phase variability, that may also optimizes other variables and explain the distribution of the data. From a computational modeling standpoint, cost-function selection is simplified by using one variable rather than weighted combinations of 2 to 3 variables. Further analysis is required to understand how minimizing relative phase variability also minimizes effort or maximizes smoothness. We added this detail in the discussion section.

Lines 738-742, Discussion: **There was indeed some overlap between maximizing stability and minimizing absolute effort or maximizing smoothness of applied force. It is therefore possible that humans optimize a weighted combination of the different variables. Further analysis is necessary to determine how maximizing stability could also lead to minimization of applied force.**

We conducted further analysis to estimate the bounds for KL-divergence. The complete KL-divergence results are shown in the RevFig. 10 below. Minimum and maximum estimates for KL-divergence for the given experimental data are indicated in the plot for reference. The minimum estimate was obtained using a Kernel Density Estimate of the data as the underlying heatmap. The maximum estimate was obtained using a randomly distributed underlying heatmap. 100 random samples were generated and the KL-divergence estimates were averaged. We added these results to the Supplementary document.

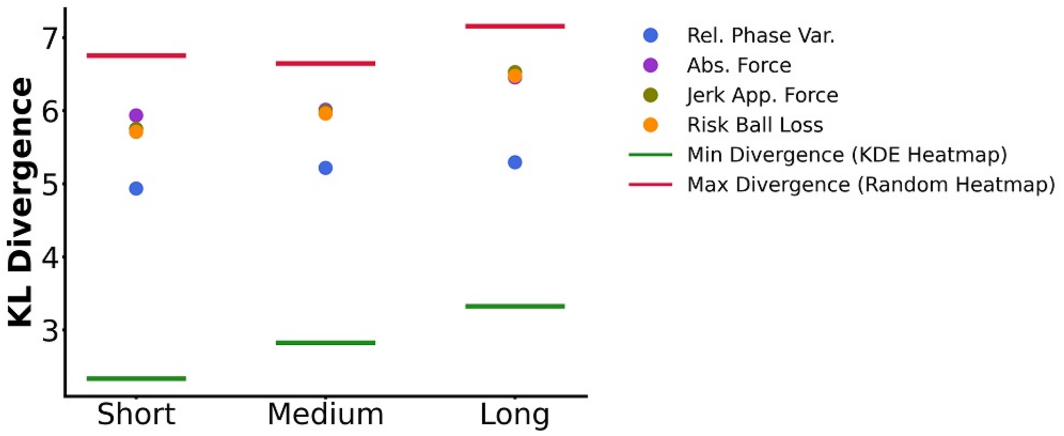

RevFig. 10 KL-divergence values for all pendulum conditions and optimization variables.

LL. 349-357: Again, more "prediction" than "hypothesis" (for same reasons stated above)...

We responded to this comment in our general response.

Fig. 6: Plots in Fig. 6A don't appear particularly "representative" – The separation between Blocked and Random appears much larger for this particular subject than the group data (Fig. 6B) might suggest.

The separation quantified as random minus blocked for the subject shown in Fig. 6A for the three pendulums are (1.97 N, 2.63 N, 1.59 N). The median separation across subjects is (2.18, 2.63, 1.99) and the mean separation across subjects is (3.24, 4.35, 4.47). These numbers show that the separation between random and blocked for this subject is not larger than the group data.

LL. 379-380: The bottom part (Rhythmic Stage) of Fig. 6B suggests at least 2 outliers...?? As the sample size for these experiments (N=12) was not especially "large" (though likely adequate) and these comparisons seem a bit close-to-marginal, it would be helpful for others trying to interpret the true strength of these findings if calculations of effect sizes were included also. I'd recommend doing this for all of the analyses performed, to the extent feasible.

We thank the reviewer for the suggestion. The effect size using the partial Eta-squared measure for the protocol factor in the rhythmic stage with outliers is $\eta^2 = 0.48$ and after removing the two outliers is $\eta^2 = 0.49$. We added the effect sizes for all effects in the manuscript. We added a sentence in the Methods to include this detail.

Lines 457-458, Methods: **We also reported the effect sizes using the partial Eta-squared (η^2) measure for reported statistical effects.**

LL. 393-408: The description given here is (1) "Methods" not "Results"... But more importantly (2) rather confusing. Was the pendulum length varied as "Gaussian noise" (L. 398), or as "a discrete random variable..." with 3 distinct values (the distribution of which might be random, but would certainly not be "Gaussian"). Likewise, LL. 403-404 states that to simulate the blocked protocols, pendulum lengths were set to 3 discrete values "with a standard deviation..." But in the experiment, the (virtual) pendulum lengths were fixed – NO variation – Yes? The experimental variation was in how the 3 pendulum lengths were presented trial-to-trial (i.e., blocked vs. random), but NOT in the lengths of the pendulum itself – Yes? Either something in the descriptions of either the experiment, the models, or both is missing, or these simulations simulated something quite different from the experiment that was conducted.
LL. 423-424: Still no explanation as to why Gaussian noise was added to the pendulum length – which (still) seems clearly contrary to the experiment conducted!...??

We thank the reviewer for pointing out this confused description and tried to clarify our congruent methods in the experiment and simulations. We used the Stochastic Optimal Open-loop Control framework to predict "feedforward" planning of impedance (Berrett and Jean, 2020; Berrett et al., 2024). Thus, we posited that humans preplan the level of impedance based on knowledge of the uncertainty in pendulum length prior to beginning a trial. It was highly likely that the impedance was also modulated through feedback processes. But the question we pursued was if such feedforward planning of impedance could explain, at least partially, the impedance results observed in our experiment.

The assumption here is that humans have knowledge of the uncertainty in pendulum length. A straightforward model of uncertainty is considering the pendulum length as a discrete random variable with uniform probabilities (Berrett et al., 2024).

We explained our modeling procedure in the methods section as follows.

Lines 359-364, Methods: **The model focused on impedance control via descending motor commands based on the knowledge of uncertainty in the dynamics of the task. We assumed that participants recognized the uncertainty in the task, preplanned impedance, and tuned the impedance in a feedforward fashion. Thus, uncertainty was added to the pendulum length to model trial-to-trial variations in pendulum length for the random protocol.**

LL. 494-496: This statement is an interpretation of the results – Not a statement of the "Results" themselves. (1) Likely best left for the "Discussion"... But also (2) Not quite correct. The model results do not (conclusively) demonstrate that participants indeed "did" the task this way... But the model simulations presented are at least "qualitatively consistent" with what participants did and so the simulations do demonstrate at least one "plausible" way in which the experimental result may have arisen. But that does not preclude the alternative possibility that other models, based on other/different criteria / assumptions etc., might not also equally (or perhaps better) replicate the main features of the data. [Again, this seems more a topic for "Discussion" than "Results"...]

We agree with the reviewer that the model presents only a plausible explanation of the participant impedance behavior. We updated the statement as follows.

Lines 661-663, Results: **Overall, these simulation results and the empirical data together suggest minimizing end-point impedance as a plausible explanation for participant's preparation and interaction strategies.**

Our results also potentially point to a physiological basis for why humans prefer stable dynamics of underactuated systems; to select a lower mechanical impedance that is also optimal for the task. We added details in the discussion section as follows.

Lines, Discussion: **Our results potentially point to a physiological rationale that humans seek stable dynamics: it may reduce mechanical impedance that also reduces effort expenditure. This leaves open the question whether stable and predictable dynamics facilitates lower mechanical impedance or whether impedance leads to stable dynamics.**

DISCUSSION:
LL. 498-522: The first 2 paragraphs offer no new information or insights – Just repetition. Could be removed, or at least greatly shortened / condensed.
LL. 524-537: See previous comment – Same here.

We have removed the first paragraph which is a repetition of motivation and the methods. However, the second paragraph summarizing the results helps to understand the subsequent discussion points.

LL. 539-547: Similar to comments regarding the Introduction – The current narratives (Intro & Discussion) don't (always) clearly articulate what is new, novel, different about *this* study relative to prior work.... Or why any such differences are "important".

In lines 514-521, we interpreted initial ball angle and cup frequency when viewed independently. We are not claiming that these findings are novel. Rather, we suggest that these results are uninteresting.

L. 563: OK. Why is this "interesting"? Is it "important" in some way? This ball-cup paradigm is nonlinear – So nonlinearities in the viable solution spaces (defined as here or possibly in other ways) would not necessarily be surprising – No?

The areas are not necessarily interesting and are expected to be nonlinear. We have updated our language as follows.

Lines 729-732, Discussion: **These areas afforded two disjunct areas that accommodated the in-phase and anti-phase solutions that subjects chose. Thus, a few participants also switched between the in-phase and anti-phase solutions with the long pendulum.**

LL. 573-575: Again, is this finding particularly surprising? Humans perform many tasks under many contexts and set of constraints (physical constraints on the task itself, external constraints like time or metronome etc. – potentially many other things). Certainly, what objectives humans choose to prioritize can and do vary depending on many different aspects of a given task.

We believe that our statement does not intend to generate surprise. We are interested in identifying control priorities that are not explicitly enforced by the task and the instruction; physical constraints, metronome, etc. Having said that, we believe identifying general control priorities irrespective of the task is important for two reasons i) explaining why humans pick specific movement solutions that are not enforced on them, ii) translating to practical applications. For example, effort expenditure is used extensively in designing prosthetics and assistive robots for neuro rehabilitation. Additionally, computational modeling of human behavior using optimization approaches requires well defined cost metrics.

LL. 575-579: This idea (such as it is?) is just not very clearly developed. It is quite hard

to discern if there is any real "conclusion" to be drawn here....

We do not discount the idea that energy costs are not prioritized when manipulating complex dynamical objects. We expect that energy costs will start to play a role upon extended performance of the task. But we deleted the last line about preparation stage which does not fit in the context of the paragraph.

LL. 580-589: This section is rather weak. First, "This finding is consistent with several previous studies..." suggests that the present work really doesn't add much "new" to what is already known in the context of this ball-cup manipulation task. Yes – Multiple prior papers from this same group working on (perhaps slightly different) variations of this task have presented quite similar conclusions.

We are not sure if 'present work' refers to all of the work in the manuscript or the portion of the work showing humans ensure stability. Nevertheless, the novelty of our work is not just to establish that humans optimize stability (Fig. 3C), but also to explain how stability is ensured by selecting preparation and interaction strategies (Fig. 4A, Fig. 5C, and Fig 5D). This has not been shown in our past work.

LL. 580-589 (Cont.): Second, there is inherent circularity in the reasoning here. In the present work "relative phase stability" was defined and quantified in terms of relative phase "variability" (Fig. 5 & LL. 344-346, etc.). By definition though, things that are "less variable" are "more predictable" and so the sentence on LL. 584-585 is a circular statement. Hence, the concluding statement that "Humans might prefer predictable dynamics..." doesn't add any new insight into the control of this task (or any other).

There is a subtle difference between stability and predictability of a system. For example, an undamped harmonic oscillator is only neutrally stable and, without perturbations, completely predictable. Predictability usually refers to future states of a system, and is facilitated by a stable system. For our argument, it is predictability that matters to the human. The statements in the paragraph would still hold if we replaced the word predictability with stability.

LL. 600-614: Again, not particularly clear. Not clear what this adds (that is new or different) to our understanding of these issues relative to several decades of prior work (that is described in this paragraph). The general conclusion (last sentence) is quite vague and highly speculative.

Indeed, the paragraph identifies the limitation that we do not analyze the finer modulation of impedance. We only present potential explanations for the modulation of mechanical impedance in our task. However, we agree with the reviewer that the statements were unclear and speculative in the current state. We refined the statements as follows:

Lines 779-786, Discussion: **One potential reason for these variations could be the coupling between the load force and grip force,(Hadjiosif and Smith, 2015; Johannson**

and Westling, 1984), i.e., the impedance can increase with the increase in the reaction force on the hand from the cup. This is consistent with the observation that increase in impedance corresponded to an increase in amplitude of the applied force during the preparation stage. Another potential explanation is that higher impedance could attenuate the unpredictable underactuated dynamics, i.e., the forces from the ball on the cup.

LL. 615-629: Again, interesting idea(s), but still somewhat vague and highly speculative [Yes – "future experiments" could be designed... But what exactly would that tell us? Why would that be "important"? Etc...]

We interpreted the increase in impedance as a means to attenuate variability, which is a view espoused by the motor control community (Sharif-Rezavian et al., 2023). In this paragraph, we considered an alternative explanation for increased mechanical impedance from the perspective of enhancing haptic perception as observed in human-human interaction experiments (Cheng et al., 2025). Experiments can be conducted to determine if participants increased mechanical impedance to better perceive the dynamics of the ball or, more generally, the underactuated dynamics. A potential experiment would manipulate the strength of haptic force feedback of the ball, e.g., by manipulating signal to noise ratio. We would predict that humans increase mechanical impedance when the reliability of force feedback is lower.

LL. 630-658: See prior comments... Sure: "Further experiments and simulations... are required..." (etc.). But to what end?
LL. 659-673: Ending (again) with "Further studies are required..." – Same question(s) – See prior comments.
LL. 674-691: This time concluding with "Its an open question whether..." – But this still leaves the same question(s) – See prior comments.

We refined some of the statements to be more specific about the aim of the proposed experiments as follows:

Lines 799-801, Discussion: **Future experiment can be designed to systematically test whether increasing impedance enhances haptic sensing of the underactuated dynamics.**

Lines 840-845, Discussion: **Our results therefore also point to a physiological rationale for humans seek stable dynamics: it may reduce mechanical impedance that also reduces effort expenditure. This leaves open the question of causality, whether stable and predictable dynamics facilitates lower mechanical impedance or whether impedance leads to stable dynamics- a question left for future research.**

METHODS:
Most all of this section largely repeats details already described in "Results" – Albeit with some additional details added here.

We have restructured accordingly.

Two general comments – First, that the narrative throughout the Methods section would be improved by being very clear which aspects of these methods were repeated from prior studies (and giving proper citations etc.)... For example: "Following [Ref. ???], we...", "Adopting the method of [Ref. ???], we..." etc. And to be very clear also where the approaches explicitly diverged from prior work... For example: "Unlike in [Ref. ???], here we..." etc.

We updated the methods section accordingly.

Second, the section on "Modeling the Cup-ball System with Uncertainty in Pendulum Length" (LL. 847-861) essentially repeats (with a few additional details) that of the Results section (LL. 393-408). It still offers no clear reason why Gaussian noise was added to the pendulum length in the simulations, when no such noise was added to the (virtual) pendulum lengths in the experiments – although it very easily could have been (since the pendulum is virtual anyway). I suspect what the authors are trying to simulate here is not the experimental task itself, but rather the participant's internal model of the task they were attempting to perform. If so, those are two very different things, and a critical distinction that is not made at all clear (either here in Methods, or before in Results).

We explained our choice of modeling in response to an earlier comment. The reviewer is correct that we assumed that participants have an internal model of the uncertainty in the task and preplan impedance to account for the uncertainty. We now explained these details in the Methods section.

Lines 359-364, Methods: **The model focused on impedance control via descending motor commands based on the knowledge of uncertainty in the dynamics of the task. We assumed that participants recognized the uncertainty in the task, preplanned impedance, and tuned the impedance in a feedforward fashion. Thus, uncertainty was added to the pendulum length to model trial-to-trial variations in pendulum length for the random protocol.**
