# Supplementary material for: Dynamical stability and mechanical impedance are optimized when manipulating uncertain dynamically complex objects
Source: PLoS Comput Biol. 2025 Dec 9;21(12):e1013797. doi: 10.1371/journal.pcbi.1013797 (PMC12704904; doi:10.1371/journal.pcbi.1013797)
Supplement: Attachment — Submitted filename: PLOS_Comp_Bio_2025__2nd_Response_to_reviewers.pdf [file pcbi.1013797.s003.pdf]

November 26, 2025

Dear Dr. Marinazzo,

Thank you for the opportunity to revise and improve our work. We would also like to thank the reviewer for their comments. We have addressed the reviewer's comments and suggestions in a point-by point-manner.

The reviewer's comments below are in bold text and our responses are in normal text format. Changes to the manuscript are in bold and blue (**bold and blue text**). The changes are reflected in the revised manuscript document in red.

Sincerely,

Rakshith Lokesh and Dagmar Sternad

EDITOR

We thank the editor for providing an opportunity to address the raised concerns. We have addressed the two major reviewer comments as follows:

1. We have explained our stand on falsifiability of experimental hypotheses. We renamed the hypotheses as predictions.
2. We explain in detail how stability is captured by relative phase variability and how predictability arises from stable dynamics. We use the term stability wherever possible to avoid any confusion.

REVIEWER 3 COMMENTS

The authors have largely done a thorough and thoughtful job of replying to the first round of reviewer comments. The manuscript has improved in many respects. There are a few points though that I think still need to be addressed and that will further improve the final manuscript. I outline those below: 1. The "So What?" (i.e., Scientific Contribution) Question: I previously raised this issue that the manuscript does not make clear what the true contribution of this new work is and/or why or how it is an "important advance" (or equivalent term) of our knowledge of these topics. I think the authors largely missed my point. In their first response to the Editor Comments, they state: "1. We continue to argue that our research topic is understudied in computational motor control compared to other research topics. While the work presented here continues our investigation into complex object control, it unifies and extends prior work..." They expand on this response in subsequent responses. I fully agree that the topic of this paper is "understudied" and agree with the authors that there is a need for more work on manipulating complex objects. I think the line of work the PI (Sternad) has developed her over the last decade plus is a strong step in that process. I agree there are not many other papers from other research groups that try to tackle these types of problems, and I agree for the same reasons the authors present. But the question I raised was not whether there was enough work being done on our field on topics like this (I agree – not enough). The question was what new contribution does *this* paper make that goes beyond merely (incrementally) "extending prior work"? This new revised manuscript still does not really answer that question clearly. That said, the question of whether these new results are "novel enough" compared to prior work is primarily an editorial decision, more so than a reviewer decision. So I will let the editors make the final call.

We believe our work unifies and extends prior work by scrutinizing the effect of dynamical uncertainty and mechanical impedance on complex object control. It significantly advances insights by: i) addressing the effect of dynamical uncertainty in complex object manipulation, ii) showing the nonlinear covariation of preparation and cup-ball interaction that ensures stability, iii) estimating mechanical impedance to reveal differential contributions for predictable and

uncertain object dynamics, and iv) accounting for our findings with a control model. We do not have any additional edits to the manuscript as we had already included the two lines of arguments for significance: 1) interaction with complex dynamical objects is understudied; and 2) this study extends our previous work in several aspects noted above.

2. Formulation of Hypotheses vs. Predictions: The authors responded by stating that "... the distinction between hypothesis and prediction is far from being agreed upon in the community." This is perhaps so, but in science in general, it has long been considered standard that "hypotheses" are only considered "scientific" if they are indeed "falsifiable" (https://en.wikipedia.org/wiki/Falsifiability). While the "hypotheses" presented here are insightful intuitive guesses (some indeed based on prior findings) as to the potential outcome of the experiment, the manuscript does not make clear if they are indeed falsifiable or not, nor what outcome/result would indicate if they were. Put another way, no viable alternative hypotheses are presented (e.g., in the form of something like "if theory A is correct, then result B is expected... Otherwise, if theory C is correct, then result D would be expected..." etc.). When the Abstract (L. 15; both previous and current) states that "The results supported... hypotheses:..." what follows is a summary of what the results *were*. The main manuscript is written in very much the same style. By any definition, a "hypothesis" is at least a *proposed* explanation (https://en.wikipedia.org/wiki/Hypothesis): i.e., it comes *before* the experiment is designed and *before* the results are known. What this manuscript presents is the exact opposite. That said, I will reiterate what (I thought) I had tried to point out in my last review – which is to say that good science (empirical science in particular) need not always be "hypothesis driven". I think this should be particularly true for journals like PLoS Computational Biology. The *computational* part of this work is why it was submitted to this journal in the first place – and it's the modeling results here that help provide a plausible a posteriori explanation of the behavior that was *observed* in the experiment. It makes not one whiff of difference here whether that observed behavior was in tow to some "hypothesis" or not. If the model can help explain that behavior, so we can better understand it... Then that's what the model is for. Period. In short – Don't try to pound a square peg into a round hole. I think this manuscript would (could) be much stronger (IMO) if all of the (misleading) "hypothesis" language were removed and the narrative focused on the modeling and the ability of the model to explain the observed behavior... Period.

In his previous comment the reviewer asked about providing better motivations and mechanisms to formulate our hypotheses. In our response, we modified the manuscript and better motivated our hypotheses (to avoid them looking like a posteriori hypotheses). In this light, we also removed one hypothesis to now have 3 instead of 4 hypotheses. In this second review, the new concern of the reviewer is the issue of falsifiability of hypotheses. We are fully aware of the Popperian emphasis on falsifiability in scientific methodology. The discussion of this claim is not new in the life sciences and also movement science (Ajemian & Hogan, 2010). We also deplore the fact that pursuing falsifiable hypotheses with alternative hypotheses is a stringent way to conduct science to obtain 'strong inference' (Platt, 1964). However, our life sciences have been

largely shaped by statistical hypothesis testing, frequentist or more recently Bayesian statistics. In our current line of experimental work, we follow this widely accepted and viable method: statistical testing of the null hypothesis. We are aware that null hypothesis testing are indeed not completely in the spirit of the Popperian view on falsifiability. However, falsification is implicit in the rejection of the null hypothesis: an experimental hypothesis is typically framed as the alternative hypothesis (H_a) that is tested against the null hypothesis (H_0). The hypothesis is statistically 'falsified' if there is enough evidence against the alternative hypothesis. Note that in statistical hypothesis testing, the hypothesis is stated in terms of operationalized variables that directly tie to the statistical test. For example, our first hypothesis stated that preparation time, a defined variable in our study, would be longer in the random protocol to establish dynamical stability compared to the blocked protocol. The null hypothesis (H_0) is that preparation times are not different between the random and the blocked protocol. The alternative hypothesis was that preparation time is greater for the random protocol than the blocked protocol. Our data did not provide enough statistical evidence to reject the alternative hypothesis.

As the reviewer suggests, the significance of our work is not hinged upon the 'hypotheses'. However, we find it useful to keep the three hypotheses as the scaffolding or backbone for our paper as it structures the presentation of our results. (Please compare to the specific aims or objectives in a research proposal.) As the reviewer has issues with the term hypothesis, we relabeled them as predictions.

3. "Stability" – "Predictability" – "Variability" Still Conflated: I previously commented on the "inherent circularity in the reasoning..." of quantifying something called "relative phase stability" by calculating the standard deviation (i.e., "variability") of relative phase and then tied these to the notion of "predictable dynamics"... The authors responded that "There is a subtle between stability and predictability..." and I completely agree! The manuscript, however, continues to conflate these terms numerous times such that any distinction between them gets completely lost. Examples: • L. 84: "By establishing stable and predictable..." – Leading to L. 90: "... to increase the predictability..." • L. 91: "Stability of a nonlinear system..." – Leading to L. 98: "... ensuring simple and predictable..." – Leading to L. 101: "... achieve stable dynamics..." • L. 137-138: "... important to stabilize the object..." – Then L. 141: "Human maximize stability..." [i.e., *not* "predictability"?] – Then L. 143: "... relative phase variability (stability)..." • L. 708-709: "... stability was assessed as the variability of..." • L. 732: "Stability Ensures Predictability..." – Leading to L. 736-737: "When the behavior... is stable, the dynamics... is more predictable..." – Leading to L. 740: "Humans might prefer predictable dynamics..." Each of these statement tries to (in some way) tie one of these terms to the other. No definitions are given and none of the "subtle differences" the authors describe in their response letter are anywhere described in the manuscript. However, any of these terms can have different meanings and/or different definitions in different contexts. In some contexts, these can be *VERY* different. For example: Center-of pressure (CoP) data during quiet standing are highly "unpredictable" (random), yet (when bounded to small amplitude, as typical for healthy adults) very "stable" (you don't fall down or even become unbalanced).... OTOH: The simple inverted pendulum model is com-

pletely "predictable" (deterministic equations of motion), but highly "unstable" (falls over at the slightest perturbation). Similarly for "variability" versus "predictability": A simple since wave (e.g., SHO) with large amplitude is completely "predictable" but also highly "variable" (large SD) (and as the authors state in their response, "neutrally stable").... The "variability" (i.e., amplitude) of the sine wave is completely independent of its "stability". OTOH: Low-amplitude white noise is minimally "variable" (small SD), but completely un-predictable (random) (but could still be considered "stable" if it doesn't grow unbounded - like the CoP example). Etc. So... It is the narrative of the manuscript itself that continues to go in circles between these three concepts without ever clearly defining any of them, or how they are either the same or different from each other. That specific definitions and distinctions are not presented in this manuscript will sow significant confusion among readers. The distinctions between these terms need to be clarified.

We thank the reviewer for pointing out the confusion of the terms stability and predictability. We agree with the reviewer that the two terms are different concepts and that they were used at times interchangeably. The confusion arose from previous work where we introduced predictability of the object dynamics as an important cost and we quantified it via mutual information (Nasseroleslami et al., 2014; Maurice et al., 2018). In subsequent studies and in this study we focused on stability (e.g., Bazzi & Sternad, 2020).

Predictability refers to and quantifies if one variable predicts behavior of another one. Stability in our work refers to the dynamical systems notion, i.e., a dynamical system can exhibit stability if it resides at a fixed point or at a limit cycle attractor (leaving a chaotic attractor aside). For the current study, where subjects oscillate the cup and ball back and forth, we hypothesize that a limit cycle attractor gets established. In line with previous experimental work on dynamic stability (e.g., Schmidt, Carello & Turvey 1990), we used variability of the relative phase between the cup and ball as a proxy for quantifying the degree of this limit-cycle stability. If two oscillating systems are synchronized and at a stable state, relative phase should be relatively constant. System noise will be low for highly stable states and high if the attractor is weaker. Hence, variability of relative phase can serve as a proxy for stability, barring more model-based stability analyses. We had defined this and referred to the relevant citations in Lines 263-265 in the Data Analysis portion of the Methods.

Considering the relation between stability and predictability, when the cup motion is viewed as the input and the ball motion as the output, the ball (output) state is more 'predictable' given the cup (input) state when the relative phase variability is low (Nasseroleslami et al., 2014; Maurice et al, 2018). Hence, highly variable relative phase indicates less predictable motion of the ball with respect to the cup. We have now further clarified the difference between stability and predictability in the manuscript. Given our experimental reliance on stability captured by variability of relative phase, we now use the term stability wherever possible to avoid any confusion.

Lines 740-742, Discussion: **For instance, in our task, when the variability of the relative phase between the cup position and ball angle is small, the ball motion is more predictable given the motion of the cup.**
